# Behavioral, Nutritional, and Genetic Risk Factors of Colorectal Cancers in Morocco: Protocol for a Multicenter Case-Control Study

**DOI:** 10.2196/13998

**Published:** 2020-01-13

**Authors:** Meimouna Mint Sidi Ould Deoula, Inge Huybrechts, Khaoula El Kinany, Hanae Boudouaya, Zineb Hatime, Achraf El Asri, Abdelilah Benslimane, Chakib Nejjari, Ibrahimi Sidi Adil, Karima El Rhazi

**Affiliations:** 1 Sidi Mohamed Ben Abdellah University Fez Morocco; 2 International Agency for Research on Cancer Lyon France

**Keywords:** diet, colorectal cancer, Morocco, case-control study, study protocol

## Abstract

**Background:**

Colorectal cancer (CRC) has been reported as the third most commonly diagnosed cancer worldwide and is currently considered as a major public health concern. A peak increase in incidence has been noted in economically transitioning countries like Morocco where industrialization started shifting from a traditional lifestyle and diet toward a more westernized diet and lifestyle.

**Objective:**

This paper aims to present the protocol of a large-scale Moroccan case-control study that aims at investigating associations of diet, other lifestyle factors, and genetic traits with CRC risk in Morocco.

**Methods:**

A case-control study was conducted between 2009 and 2017, including 3032 case-control pairs (1516 cases and 1516 controls) matched on sex, age, and center in 5 major public health hospitals in Morocco. Questionnaires on sociodemographic data, lifestyle, family history of CRC, and nonsteroidal anti-inflammatory drugs (NSAIDs) were completed by trained investigators during face-to-face interviews. In addition, participants completed a semiquantitative food-frequency questionnaire, developed to assess food intake in the Moroccan population. Information regarding genetic factors was recorded for cases, and paraffin blocks (with embedded tumor tissues) are available in 3 collaborating hospitals. Conditional logistic regression analysis is planned to assess associations between diet and CRC risk. Binary logistic regression is considered to predict associations between mutations and nutritional risk factors including only CRC case series.

**Results:**

Altogether, 2966 cases-control pairs (1483 cases and 1483 controls) were considered eligible and included in this study. Both cases and controls did not differ significantly with respect to age (*P*=.36), sex (*P*=.51), center (*P*>.99), marital status (*P*=.30), and NSAID use (*P*=.08). However, participants in the control group were significantly more likely to have a high income level and live in urban areas and to have a high level of education than cases.

**Conclusions:**

This is the first study investigating potential risk factors of CRC such as lifestyle, diet, and genetic factors, originating from a southern Mediterranean country with low but increasing CRC prevalence. Identified risk factors allow the establishment of evidence-based preventive actions regarding nutrition and other lifestyle habits adapted to the Moroccan context. In brief, this study will promote cancer research and prevention in Morocco.

**International Registered Report Identifier (IRRID):**

RR1-10.2196/13998

## Introduction

### Background

Colorectal cancer (CRC) is the third most common cancer in men and the second most common cancer in women worldwide [1,2]. The high CRC incidence and magnitude make it a real public health concern [3]. Therefore, it is essential to determine its risk factors as a basis for evidence-based prevention strategies. The risk factors of CRC are complex and involve genetic and environmental factors [4,5]. Indeed, 30% to 70% of CRCs are considered to be because of environmental factors such as lifestyle and eating habits [6-8].

According to the recent World Cancer Research Fund (WCRF)/American Institute for Cancer Research (AICR) 2018 report, high red and processed meat intake, high body fatness, high consumption of alcoholic drinks, and low levels of physical activity have been associated with increased CRC risk, whereas diets high in antioxidants, in particular fruits and vegetables, or fibers have been associated with reduced risk [9]. A high body mass index is associated with an increased risk of occurrence of large adenomas at the level of the colon and rectum [10].

CRC incidence is indeed particularly high in Western countries with high meat consumption, such as Australia and the United States [11], whereas lower in Mediterranean countries [1] which could potentially be attributed to the quality of the Mediterranean diet, including high consumption of fruits, vegetables, and olive oil [12].

Regarding genetic factors, epidemiological studies conclude that the risk of developing CRC increases with increasing first-degree family history [13]. Several recent studies attempt to show a potential interaction between genetic predisposition and dietary factors [14]. One of these interactions could involve the acetylator status [15]. Indeed, rapid acetylating subjects (rapid phenotype of N acetyl transferase [NAT2]) are more likely to develop CRC, particularly when consuming overgrilled meat, through greater activation of heterocyclic amines [15]. Another example demonstrating the importance of considering genetic status is the analysis of certain mutations that occur in the early stages of CRC such as Kirsten rat sarcoma (KRAS) and proto-oncogene B-Raf (BRAF) mutations. These mutations were associated with certain foods, nutrients, and micronutrients, and it has been suggested that the risk of KRAS and BRAF mutations may depend on dietary and lifestyle factors [13,16].

The majority of studies investigating CRC risk factors have been conducted in countries with high CRC prevalence [11]. Results from these high-prevalence studies are sometimes controversial and not always applicable to low-prevalence countries such as Morocco, which have started feeling the effects of the triple demographic, epidemiological, and nutritional transition [17-19].

According to the cancer incidence data from Globocan 2018, CRC ranks as the third most common cancer in Morocco after lung and prostate cancer in men and after breast and cervical cancer in women (8.7% and 7% of all types of cancer, respectively). Furthermore, this number of new cancer cases seems to increase each year [20,21]. According to the Cancer Registry of the Greater Casablanca Region (2005 to 2012), age-standardized incidence rates raised from 3.8 to 8.4 and from 2.6 to 7.4 per 100,000 in Moroccan men and women, respectively [20,21]. At the same time, cancer risk factors and behaviors of the Moroccan population are progressing rapidly owing to profound societal and industrial changes. This progression would also be linked to the nutritional transition that shows local specificities so far scarcely documented [22].

Moroccan food habits are characterized by their tradition and culture but also by profound changes to a Western lifestyle [22]. Increasingly, the westernization of food fads that is characterized by more consumption of red meat, processed meat, alcohol, and *junk food* is observed, all this in a context of a sedentary and stressful lifestyle [11]. Given that the majority of these changes are considered to be CRC risk factors, this could explain part of the increasing incidence rates in a country supposed to be low in CRC incidence.

### Objective

In Morocco, to our knowledge, no previous study has investigated associations between these risk factors and CRC. Therefore, we designed a multicenter case-control study to evaluate the relationship of the Moroccan diet, physical activity, and other lifestyle habits with CRC risk. This study will also describe the genetic profiles of CRCs in Morocco and their interaction with food intakes. Finally, we will also select CRC index cases that would identify family cancers in Morocco.

## Methods

### Design

**Figure 1:**
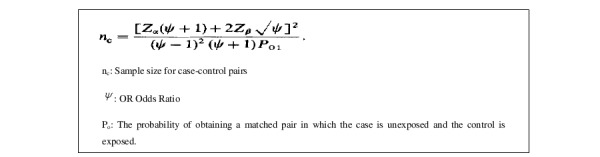
Formula specific for individual-matched case-control studies.

This case-control study was conducted between September 2009 and February 2017 in 5 major public health hospitals in Morocco, namely, the University Hospital Center (UHC) Hassan II of Fez, UHC Ibn Sina of Rabat, UHC Mohammed VI of Oujda, UHC Ibn Rushd of Casablanca, and UHC Mohammed VI of Marrakech. The sample size estimation was based on red meat consumption as one of the main exposures of interest. According to the National Survey of Dietary Habits in Morocco, the proportion of Moroccan adults eating red meat at least twice a week was 62.7% [23]. The sample size was calculated using the following formula specific for individual-matched case-control studies (Figure 1) [24], considering a type I error (Cronbach alpha) equal to 5%, a statistical power of 90% (beta=.10), and a minimum difference in terms of risk of 43% as reported by the WCRF/AICR report [5].

The number of pairs required for the study was 1496, that is, in each of the 2 groups 1496, which was rounded to 1500.

### Definition of Cases and Controls

Cases were patients who had newly confirmed CRC diagnosis by histopathology less than 3 months before the interview and who did not start any therapeutic protocol (chemotherapy, radiation therapy, hormone therapy, or surgery) yet at the time of inclusion. However, for some patients included in the study, the diagnosis was done at the time of surgery. For the exclusion criteria, patients who had received chemotherapy, radiation therapy, or hormone therapy were excluded as they were not considered as newly diagnosed, and the therapy may affect their lifestyle habits.

Each case was matched with a control subject by age (±5 years), sex, and center. Other eligibility criteria included the following: at least 18 years old, no prior history of diabetes mellitus, capability to give consent, and ability to communicate and carry out the interview. Extra exclusion criteria were patients under exclusive palliative treatment and patients confined to their chairs or beds.

Controls were selected from the same local population and in the same hospitals as cases, among healthy subjects accompanying other patients or visitors. Eligibility criteria were the same as for cases, except that the controls should not have any personal history of CRC or any other type of cancer. Unfortunately, we did not have the funding to carry out a fecal occult blood test or colonoscopy for controls. However, to avoid (or minimize) the inclusion of false controls, questions about their medical history and clinical symptoms related to CRC were systematically asked to all controls.

### Data Collection

Data were collected through face-to-face interviews conducted by trained investigators. All participants were asked to answer questionnaires on the following topics.

#### Sociodemographic Information

It includes age (obtained from personal identification numbers); sex; center (Rabat, Marrakech, Fez, Oujda, and Casablanca); residency (urban and rural); profession (employed, retired, unemployed, housewife, and student); marital status (single, married, divorced, and widow[er]); education level (illiterate, primary, secondary, and higher); income level (<2000 MAD [Moroccan dirham], 2000-5000 MAD, >5000 MAD); and type of habitat (luxurious habitats, new medina, slum habitats, modern habitat, and old medina).

#### Clinical Data

Clinical data were collected regarding their CRC diagnosis (the type of cancer, the date of biopsy, the stage of cancer, operated patient, and date and type of surgery), family history of CRC in first- and second-degree relatives, and the use of nonsteroidal anti-inflammatory drugs (NSAIDs).

#### Substances Use

It includes alcohol consumption and smoking status. Alcohol consumption was divided into 2 categories: never and current consumers. The current consumers were asked to precise the quantity and the frequency of consumption of the 5 proposed types of alcoholic beverages (red wine, white wine, pink wine, whisky, and vodka). Smoking status was defined according to the International Union Against Tuberculosis and Lung Diseases Guide [25]. Respondents were defined as current smokers (daily and occasional smokers) if they were smoking at the time of the survey and had smoked more than 100 cigarettes in their lifetime. They were defined as ex-smokers if they had smoked more than 100 cigarettes in their lifetime but stopped smoking during the past more than 3 months at the time of the survey; and they were defined as never smokers if they had never smoked or had smoked less than 100 cigarettes in their lifetime [25]. Thus, smoking status was divided into 3 categories: never smokers, ex-smokers, and current smokers.

#### Physical Activity Levels

To assess the physical activity levels, participants were asked the average time they spent on the following activities during the past year: physical activity at work, travel to and from home, and recreational activities. According to the Global Physical Activity Questionnaire [26], the number of hours per week spent engaging in each activity was multiplied by the corresponding energy expenditure, expressed as metabolic equivalent of task (MET), and the product was taken as the physical activity score expressed as MET-minutes per week. The intensity of physical activity was classified into 3 categories: light intensity (<600 MET-minutes per week), moderate intensity (600-3000 MET-minutes per week), and vigorous intensity (≥3000 MET-minutes per week).

#### Anthropometric Measurements

The anthropometric measurements included height and current weight at the time of the survey and have been extracted from medical records, whereas all previous measurements (before the appearance of the symptoms), waist, and hip sizes were self-reported.

### Dietary Data

Dietary data were collected using a validated semiquantitative Food Frequency Questionnaire (FFQ) that was developed to assess food intake in the Moroccan population [27]. It was inspired from the Global Allergy and Asthma European Network (GA^2^LEN) [28] and validated in the Moroccan context [27].

Multimedia Appendix 1 depicts this FFQ that included 255 foods and the following 32 food groups: (1) bread, (2) breakfast with grains, (3) couscous (one of the traditional staple foods of Maghreb countries’ cuisine prepared by durum wheat semolina), (4) pasta, (5) cake, (6) rice, (7) sugar, (8) sweets without chocolate, (9) chocolate, (10) vegetable oil, (11) margarine and vegetable fat, (12) butter and animal fat, (13) dried fruit, (14) legumes, (15) vegetables, (16) potatoes, (17) fruits, (18) juice, (19) nonalcoholic beverages, (20) coffee/tea, (21) beer, (22) wine, (23) other alcoholic beverages, (24) red meat and processed meat, (25) poultry, (26) sekat (offal and brain), (27) fish, (28) eggs, (29) milk of cow/soya, (30) cheese, (31) other dairy products, and (32) miscellaneous foods (Multimedia Appendix 1).

Participants, both cases and controls, recorded their food consumption of the past year before the interview. Frequency of food consumption was recorded in 8 different categories (never, 1-3 times per month, once a week, 2-4 times per week, 5-6 times per week, once a day, 2-3 times per day, and ≥4 times/day). Regarding seasonal foods, participants were asked to answer the question based on intakes during periods/seasons when these foods are available. The daily intake of the foods was then calculated according to the number of months per year that each seasonal food was available. To convert foods into nutrient intakes, we essentially used food composition data from Tunisia [29] and Morocco [30]. The nutrient and energy intakes were calculated by multiplying the daily intakes of each food item by the nutrient and calorie content (per 100 g) of all food items.

In addition, the reproducibility and validity of this FFQ were evaluated among 105 healthy Moroccan adults. The results showed a good relative validity (deattenuated correlations ranging from 0.24 for fiber to 0.93 for total monounsaturated fatty acids) and a good reproducibility (intraclass correlation coefficient ranging from 0.69 for fat to 0.84 for Vitamin A) [27].

### Genetic Data

Pathology tumor samples were collected for 170 patients, whose anatomopathological tests were done in the UHCs and whose block tumors were available. In fact, the majority of patients are used to do their biopsies outside the university hospitals, in private sectors, which are difficult to access and not always willing to participate in research projects. For this reason, biopsies done in the private sector were not included in this study. In addition, only 3 out of 5 public health hospitals provided consent to participate in the genetics part of the study and because of financial difficulties, only a subsample of pathological tumor samples was obtained. The tumor samples were collected between February 2016 and July 2017 from 3 public health hospitals in Morocco (Casablanca, Oujda, and Rabat). In addition, all molecular analyses were done in the laboratory of genetics at the University Hospital Hassan II in Fez. A pathologist doctor classified the samples embedded in paraffin and registered and coded using consecutive and unique identification numbers. These stored paraffin-embedded tissues were collected from the 3 UHCs included in this study. DNA was extracted using an Invitrogen RNA/DNA isolation kit by manually scraping tissue from unstained slides. The BRAF and KRAS mutations will be determined by direct sequencing and analyzed by methylation-specific polymerase chain reaction.

### Data Cleaning and Handling

In total, the study recruited 3032 subjects (1516 cases and 1516 controls). Table 1 depicts the data cleaning and handling by reporting the exclusions. Exclusions before starting statistical analysis included participants with unspecified primitive cancer (n=7), cases with old biopsies (6 cases), participants with missing dietary data because the FFQ was not well filled (n=10), duplicate records (n=2), and unmatched records (n=8).

**Table 1:** Exclusions of study participants (1516 cases and 1516 controls invited in the study) during data cleaning and handling (N=1516).

Exclusion criteria^a^	Excluded cases, n (%)	Excluded controls, n (%)
Unspecified cancer	7 (0.46)	0 (0.00)
Patients with old biopsies	6 (0.39)	0 (0.00)
Food-Frequency Questionnaire empty	10 (0.65)	10 (0.65)
Duplicate records	2 (0.13)	0 (0.00)
Unmatched cases/controls	8 (0.52)	8 (0.52)
Total	33 (2.17)	18 (1.18)

^a^Total individual matching (included in the study): 1483 cases and 1483 controls.

### Participation Rate

The participation rate in this study was 97% (1516/1555) for cases and 0.75% (1516/2000) for controls. Table 2 depicts the missing data of all variables for the final sample included in this study (1483 cases and 1483 controls).

**Table 2:** Missing data for each variable (1483 cases and 1483 controls).

Variables	Subjects with missing information	
	Cases (N=1483), n (%)	Controls (N=1483), n (%)
Surgery type	1 (0.06)	0 (0.00)
Surgery date	18 (1.21)	0 (0.00)
Number of years of study	336 (22.65)	408 (27.51)
Current weight	8 (0.53)	15 (1.01)
Waist size	680 (45.85)	713 (48.07)
Hip size	1019 (68.71)	1006 (67.83)
Family history of colorectal cancer but type of family relationship unknown	2 (0.13)	1 (0.06)
Nonsteroidal anti-inflammatory drug use	2 (0.13)	3 (0.20)
Personal address	27 (1.82)	20 (1.34)
Heading of food groups	47 (3.16)	48 (3.23)
Cereals	15 (1.01)	5 (0.33)
Oils	4 (0.26)	2 (0.13)
Butter	1 (0.06)	0 (0.00)
Nonstarchy vegetables	3 (0.20)	9 (0.60)
Starchy vegetables	4 (0.26)	2 (0.13)
Soft drink	9 (0.60)	13 (0.87)
Tea	1 (0.06)	3 (0.20)
Coffee	1 (0.06)	1 (0.06)
Red meat	9 (0.60)	13 (0.87)

### Ethics and Availability of Data

The protocol of this study has been reviewed and approved by the ethics Committee at the University of Fez in September 2009. Written informed consent was obtained from all participants before enrollment. Confidentiality of data is secured by removing personal identifiers from the datasets.

### Statistical Analysis

The descriptive information of the categorical variables was presented as the frequency of each category and for the continuous variables by means and standard deviation. Differences between continuous variables were examined by using the student *t* test (2 tailed) for matched samples. Chi-square tests (McNemar) were used to examine differences among categorical variables.

## Results

As of February 2017, we enrolled 2966 cases and controls (1483 cases and 1483 controls) considered eligible and included in our study. To date, the genetic part of the study is still ongoing. Table 3 shows the sociodemographic variables and lifestyle factors among CRC cases and controls. Both cases and controls did not differ significantly with respect to age (*P*=.36), sex (*P*=.51), center (*P*>.99), marital status (*P*=.30), and NSAID use (*P*=.08). However, participants in the control group were significantly higher educated than cases.

**Table 3:** Main characteristics of cases and controls in the Moroccan colorectal cancer case-control study.

Characteristics			Cases (N=1483)	Controls (N=1483)	*P* value^a^
**Matching variables**					
	Age at recruitment (years), mean (SD)		56.45 (13.98)	55.51 (13.73)	.36
	**Sex, n (%)**				
		Female	746 (50.30)	746 (50.30)	.51
		Male	737 (49.69)	737 (49.79)	—^b^
	**Center, n (%)**				
		Rabat	482 (32.50)	482 (32.50)	—
		Marrakech	27 (1.82)	27 (1.82)	>.99
		Fes	241 (16.25)	241 (16.25)	—
		Oujda	251 (16.92)	251 (16.92)	—
		Casablanca	480 (32.36)	480 (32.36)	—
**General characteristics**					
	**Residency, n (%)**				
		Urban	1021 (68.84)	1117 (75.32)	.001
		Rural	462 (31.15)	366 (24.67)	—
	**Marital status, n (%)**				
		Single	142 (9.57)	146 (9.84)	—
		Married	1128 (76.06)	1138 (76.73)	.30
		Divorced	47 (3.16)	59 (3.97)	—
		Widow(er)	166 (11.19)	140 (9.44)	—
	**Education level, n (%)**				
		Illiterate	936 (63.11)	748 (50.43)	—
		Primary	281 (18.94)	276 (18.61)	.001
		Secondary	178 (12.00)	271 (18.27)	—
		Higher	88 (5.93)	188 (12.67)	—
	**Income level (Moroccan dirham), n (%)**				
		<2000	1216 (81.99)	1061 (71.54)	—
		2000-5000	208 (14.02)	299 (20.16)	.001
		>5000	59 (3.97)	123 (8.29)	—
	**Family history of colorectal cancer, n (%)**				
		Yes	83 (5.59)	12 (0.80)	.001
		No	1400 (94.40)	1471 (99.19)	—
	**Past regular nonsteroidal anti-inflammatory drug use, n (%)**				
		Yes	105 (7.08)	126 (8.49)	.08
		No	1378 (92.91)	1357 (91.50)	—

^a^Differences between continuous variables were examined by using student *t* test. Chi-square tests (McNemar) were used to examine differences among categorical variables.

^b^Not applicable.

Compared with controls, cases were more likely to have a family history of CRC. At last, cases were slightly but not significantly older than controls (mean 56.45 years, SD 13.98 years vs mean 55.51 years, SD 13.73).

## Discussion

The primary aim of this study was to evaluate the relationship of diet, physical activity, and other lifestyle habits with CRC risk in Morocco. To our knowledge, this is the first study designed to investigate this association in the Moroccan context. Similar studies have been conducted mostly in western countries [31-34] and few of them in some countries of the Middle East and the Northern African (MENA) region, 2 regions that are culturally quite similar to Morocco.

The studies conducted in the MENA region had low sample sizes, and their results could not be considered as representative. In addition, cases were not necessarily newly diagnosed with CRC [35,36]. This might affect the reliability of the results as cases may change their dietary habits after diagnosis. Moreover, the control groups were selected among patient’s visitors, and the authors of these studies did not check for familial relationships between cases and their matched controls, what could potentially introduce selection bias and overmatching problems. Besides, all these case-control studies [37-42] used matching methods to control for some confounding factors such as age and sex. Nevertheless, they did not use the model of conditional regression for statistical analysis, which is highly recommended for this type of study design [43]. At the international level, many case-control studies have been conducted in Western countries with strong methodologies and great statistical power [44,45]. These reported strong evidence for the association between some foods and CRC risk, such as red and processed meat and dairy products [46-48]. Other associations are either controversial or not approved yet [10,49]. Furthermore, all these western studies did not include dietary habits from other regions of the world including the MENA region or Morocco. Moreover, the results drawn from some strong associations found for some foods, such as pork and alcohol [32,44], may not be applicable to the Moroccan context, where these types of foods are not commonly consumed for cultural or religious reasons. Therefore, this Moroccan case-control study checks whether or not the CRC-related factors are the same as in western countries. It also describes the specificities of Moroccan food habits in relation to CRC risk in Morocco.

This study has some potential limitations. The major one may be the recall bias that is known to be related to any retrospective study and was minimized in this study through the enrollment of the newly diagnosed patients who presumably remember their eating habits just before the onset of their illness better than after therapy or at more advanced cancer stages. The lengthy recall of dietary information can be considered as the second limitation. However, it could also be considered as an advantage of the study that very detailed dietary intake data were collected and available for statistical analyses; conversely, short dietary questionnaires may underestimate the true variation in food intake. Furthermore, the measurement error associated with the FFQ is another possible limitation of our study. To minimize the effect of these biases and errors and to avoid the likely influence of the lengthy recall of dietary information on data quality, interviewers were trained to help participants to fill in the questionnaire by clarifying questions if needed, which may increase the accuracy of answers. In addition, the dietary questionnaire has been validated, showing moderate to good validity.

The use of visitors as controls is another potential limitation of the study. In fact, recruitment of controls from outside the hospital setting was not feasible. Thus, we selected healthy subjects accompanying other patients or visitors as controls. As a condition of recruitment to avoid bias related to such controls, visitors must not be a relative or the patient’s partner. We also made sure that they did not have the same family history and did not live in the same circumstances. The majority of those recruited were friends or neighbors of the patients.

The major strength of this study is that it is the first of its kind investigating potential risk factors of CRC such as lifestyle, diet, and genetic factors, originating from a southern Mediterranean country with low but increasing CRC prevalence. Its multicentric design and its large sample allow us to describe the national food habits as well as the epidemiological profile of Moroccan CRC cases. Moreover, dietary data were collected through a validated FFQ [27], in addition to a large battery of other lifestyle behaviors.

Identified risk factors will allow the establishment of evidence-based preventive actions regarding nutrition and other lifestyle habits tailored to the African, more particularly Moroccan, context. To conclude, this study will promote cancer research and prevention in Morocco.

## Appendix

Multimedia Appendix 1 Foods included in the validated Food Frequency Questionnaire (FFQ) for Morocco.

## Abbreviations

AICR: American Institute for Cancer Research
BRAF: proto-oncogene B-Raf
CRC: colorectal cancer
FFQ: Food Frequency Questionnaire
KRAS: Kirsten rat sarcoma
MENA: Middle East and the Northern African
MET: metabolic equivalent of task
NSAID: nonsteroidal anti-inflammatory drug
UHC: University Hospital Center
WCRF: World Cancer Research Fund
