# Supplementary material for: Behavioral, Nutritional, and Genetic Risk Factors of Colorectal Cancers in Morocco: Protocol for a Multicenter Case-Control Study
Source: JMIR Res Protoc. 2020 Jan 13;9(1):e13998. doi: 10.2196/13998 (PMC7006499; doi:10.2196/13998)
Supplement: Multimedia Appendix 1 [file resprot_v9i1e13998_app1.pdf]

Multimedia Appendix

Table 1 Foods included in the validated Food Frequency Questionnaire (FFQ) for Morocco

Name
1. Bread
Any type of bread
Bread, whole meal, average (Durum wheat)
Bread, white, French stick
Bread of zouane (Rye)
Mllaoui/rghaif/mssemen/batbout/matlouaa
Bread of smida/harcha (semolina)
Homemade bread
Other type of bread (barley)
2. Breakfast with grains
Any type of grains
Assida/smida
Dchicha/belboula
Porridge (herbel), mflak
All-Bran
Corn flakes
3. Couscous
Barley couscous, cooked with meat, vegetables, and dried grapes

Barley couscous, cooked with sugar and cinnamon<br>Wheat couscous, cooked with meat, vegetables, and dried grapes<br>Wheat couscous, cooked with sugar and cinnamon<br>Corn couscous, cooked with meat, vegetables, and dried grapes<br>Corn couscous, cooked with sugar and cinnamon
4. Pasta
Any type of pasta<br>Pasta white boiled (spaghetti, macaroni)<br>Pasta, whole meal, boiled<br>Pasta with meat, vegetables, and cheese<br>Chaaria Mhammsa
5. Cake
Any type of cake, cherry<br>Madeleine cake<br>Cake with dates<br>Croissants<br>Moroccan sweets<br>Basboussa Maqrout<br>Aassida<br>Doughnuts, ring<br>Rice pudding, canned

Pancake roll<br>Cake, coconut<br>Sellou Zammita<br>Chabbakia Mkharrka
6. Rice<br>Any type of rice, brown, boiled<br>Rice, white, easy cook, boiled<br>Rice, brown, boiled<br>Noodles, rice, dried
7. Sugar<br>Sugar, white<br>Jam, fruit spread<br>Honey<br>Syrup, golden
8. Sweets without chocolate<br>Chew sweets<br>Fudge<br>Toffees<br>Cereal chewy bar<br>Polo skimo glace
9. Chocolate

Any type of chocolate<br>Chocolate-covered bar with fruit/nut/ biscuits<br>Natural white and black chocolate
10. Vegetable oil
Oil, vegetable, blended, average<br>Oil, safflower<br>Oil, olive<br>Oil, Argan<br>Oil, corn
11. Margarine and vegetable fat
Any margarine and vegetable fat (except soya fat)<br>Light margarine or less fat (30% fat)<br>Margarine (from 40% to 60% fat)<br>Normal margarine (more than 70% fat)<br>Mixed fat (except soya)<br>Original fat of soya (any type)
12. Butter and animal fat
Any animal fat (butter)<br>Butter with less fat (<40%)<br>Butter with less fat (from 40 to 60% fat)<br>Smen (traditional butter)

13. Nuts
Any type of dried Fruit
Peanuts, plain
Cashew nuts, roasted and salted
Almonds, roasted
Walnuts
Pistachio nuts, roasted and salted
Chestnuts
Oak nut
14. Legumes
Any legumes
White beans, boiled
Lentils, red, split, boiled
Chick peas, whole, dried, boiled unsalted
Green beans/French beans, raw
Broad beans, frozen, boiled in unsalted
Soya beans, dried, boiled
Peas, raw
15. Vegetables (mean dish)
Any vegetables except potatoes
Lettuce, average, raw

Spinach, raw
Fenugreek seeds
Rejla; Bakkoula
Mloukhia (Jews Mallow)
Tomatoes, raw
Aubergine, raw (Eggplant)
Courgette, raw (squash)
Peppers, red, raw, yellow
Cucumber, raw
Carrots, raw
Parsnip, raw
Swede, raw
Artichoke globe, raw
Radish, white, mooli, raw
Beetroot, raw
Chili peppers, green, raw
Sweet corn kernels, raw
Asparagus, raw
Aromatic herbs (mint basilica, parsley basil coriander)
Leeks, raw
Mushrooms, black, white

Onions, raw<br>Garlic, raw<br>Cauliflower, raw<br>Pumpkin red<br>Brussels sprouts, raw<br>Broccoli, green, raw<br>Cabbage white, red, green<br>Tomatoes stuffed with vegetables<br>Pickle, mixed veg<br>Ginger, root
16. Potatoes (mean dish)
Any type of potatoes<br>Potatoes, old mashed with hard margarine<br>Potatoes, old, baked, flesh and skin<br>Chips, homemade, fried in blended oil<br>Salad, potato with French dressing<br>Potato cakes fried in vegetable oil<br>Tortillas<br>Sweet potato
17. Fruits (one unit)
Any type of fruits

Apples
Pears
Bananas
Peaches
Avocado
Cherries
Lemon pickles
Mulberries, raw, blackcurrants, raspberries
Watermelon
Grapes
Mangoes
Apricots
Nectarines
Plums
Dried mixed fruit
Pineapple
Kiwi fruit
Juice, lemon
Oranges
Mandarin
Grapefruit

Fruit cocktail, conserved in syrup<br>Figs, raw, dried<br>Black or green olives<br>Raisins<br>Dates, dried with stones
18. Juice<br>Orange juice (concentrate)<br>Pomegranate juice (pomegranate, raw)<br>Any other type of juice
19. Nonalcoholic beverages<br>Lemonade<br>Beet juice<br>Mineral water
20. Coffee/tea<br>Tea, infusion<br>Coffee, instant, made up<br>Zizwa (coffee, liquid)<br>Tea, Chinese, leaves, infusion<br>Mint, fresh<br>Other herbal infusions

21. Beer
Any type of beer
**22. Wine**
Any type of wine
Wine, red
Wine, white, dry
Wine, rose
**23. Other alcoholic beverages**
Port, sherry, liqueur,
Spirits 37.5%
**24. Red meat**
Any type of red meat (beef, cow, lamb, goat)
Beef, fillet steak, forerib, lean & fat, roast, steamed, grilled
Beef in tagine
Minced meat of beef
Lamb, grilled, steamed, roasted
Lamb cooked in tagine, mrouzia
Minced meat of lamb
Goat meat
Veal, fillet, roast
Camel meat

Rabbit, duck, partridge
Sausage of beef, lamb, cow, chilled, fried
Kocha or bread filled with meat
Kabab, shawarma
Pork
Khliaa/dried meat
Khliaa (dried meat with salt and cooked with fat), cow
Khliaa (dried meat with salt and cooked with fat), sheep
Qaddid (dried meat with salt), cow, sheep
Dried pork meat
25. Poultry
Any type of chicken
Chicken steamed
Chicken cooked in tagine
Chicken grilled and roasted
Turkey steamed
Turkey cooked in tagine
Turkey grilled and roasted
Sausage and skewer of turkey
Poultry smoked, conserved
Any poultry smoked, conserved (eg, mortadella and casheer)

26. Offal (sekat)
Liver of beef, lamb
Tongue, heart, kidney, head, brain, of cow or beef or sheep, lamb
27. Fish
Any fish fresh, smoked, white, fat
Fresh fat fish (eg, salmon, tuna, truite, sardine, and bouri)
White fresh fish (eg, sole and merlan)
Fresh fish/other sea foods (eggs of fish)
Seafood shrimp, squid, mussels
Frozen seafood
Frozen fat fish (eg, salmon, tuna, truite, sardine, and bouri)
Frozen white fish (eg, sole and merlan)
Conserved fat fish (eg, salmon, tuna, truite, sardine, and bouri)
Fat fish dried or smoked (eg, salmon, tuna, truite, sardine, and bouri)
White fish dried or smoked (eg, sole and merlan)
Conserved seafood shrimp, squid, and mussels
28. Eggs
Farmer eggs
Farmer egg boiled or sandwich
Farmer eggs' meals: omelet, eggs with tomatoes, eggs with pepper and tomatoes
Dessert with farmer eggs (cake, egg tart)

Industrial eggs<br>Industrial egg boiled or sandwich<br>Industrial eggs' meals: omelet, eggs with tomatoes, eggs with pepper and tomatoes<br>Dessert with industrial eggs (cake, egg tart)
29. Milk of cow/milk of soya<br>Whole milk (milk, cow, whole, 3.5% fat)<br>Lben (alone or with fruit)<br>Skimmed milk (milk, cow, skimmed, 0.5% fat)<br>Semi skimmed milk (milk, cow, partly skimmed, 1.5% fat)<br>Milk free fat<br>Raib<br>Soya milk<br>Saykook<br>Yogurt<br>Yogurt Activia<br>Soya yogurt
30. Cheese<br>Any type of cheese<br>Hard cheese (eg, cheddar and parmesan)<br>Soft cheese (camembert, brie, Philadelphia)<br>Semihard Cheese (gouda, emmental/edam)

Jben (natural or aromatic)<br>Fresh cheese (eg, vita, mozzarella)<br>Others: La vache qui rit, Kiri, Coeur du lait, Junior
31. Other dairy products
Ice cream<br>Cream<br>Fresh cream<br>Double cream
32. Miscellaneous foods
Soup with vegetables and meat<br>Soup with vegetables and grains (eg, Dchicha and Smida)<br>Soup with meat or offal<br>Soup with fish<br>Tagine with meat or poultry<br>Salt brick<br>Pizza<br>Sorghum<br>Chili sauce<br>Ketchup<br>Salad sauce<br>Mayonnaise

Mustard
